# Métastases cutanées révélant un carcinome indifférencié du nasopharynx (UCNT)

**DOI:** 10.11604/pamj.2017.26.139.11917

**Published:** 2017-03-14

**Authors:** Jamal Fatihi, Salah Bellasri

**Affiliations:** 1 Service de Médecine Interne, 5^iéme^ Hôpital Militaire de Guelmim, Mohammed V University in Rabat, 81000 Guelmim, Maroc; ^2^Service de Radiologie, 5^iéme^ Hôpital Militaire de Guelmim, Mohammed V University in Rabat, 81000 Guelmim, Maroc

**Keywords:** Cancers du nasopharynx, UCNT, métastases cutanées, Cancers of the nasopharynx, UCNT, cutaneous metastases

## Image en médecine

Les cancers du nasopharynx, dominés par les carcinomes épidermoïdes surtout de type undifferenciated carcinoma of nasopharyngeal type (UCNT), sont des tumeurs particulières au sein des autres cancers de la sphère ORL par leurs évolutivité, le haut potentiel métastatique à distance et la survenue chez des sujets jeunes sans facteurs de risque classiques alcoolo-tabagiques. Les métastases cutanées révélatrices des carcinomes UCNT sont extrêmement rares. Nous rapportons le cas d'un homme de 50 ans, sans passé pathologique particulier, hospitalisé pour des nodules sous cutanés (cuir chevelu) (A), région lombaire (B) et flanc droit (C)) apparus trois mois auparavant dans un contexte d'altération de l'état général et de douleurs osseuses. La biopsie exérèse avec étude anatomopathologique d'un des nodules était en faveur d'une localisation secondaire d'un carcinome UCNT. L'endoscopie nasale mettait en évidence un processus bourgeonnant endonasal (D) dont la biopsie confirmait le type carcinomateux UCNT. Le bilan d'extension classait la tumeur en stade IV C avec métastases cutanées, ganglionnaires, et osseuses. L'évolution était défavorable après 9 mois malgré la radiochimiothérapie.

**Figure 1:**
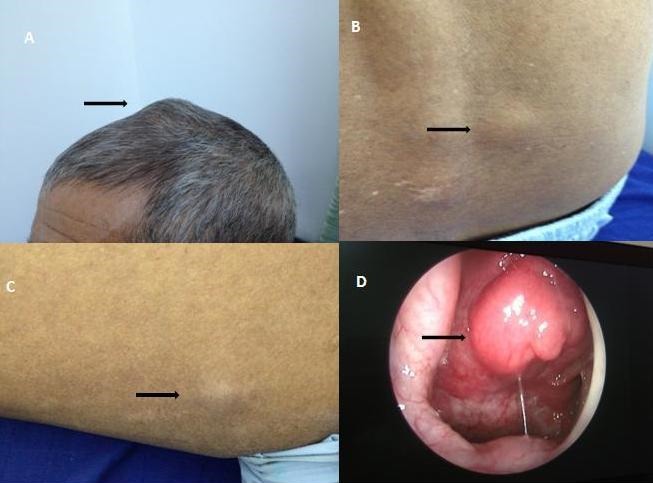
A) nodule sous cutané du cuir chevelu; B): nodule sous cutané para vertébral droit; C) nodule sous cutané du flanc droit; D) processus tumoral bourgeonnant endonasal

